# Challenges and Strategies for Improving Training of Mid-Level Research Personnel in Nigeria

**DOI:** 10.5334/aogh.2405

**Published:** 2019-06-18

**Authors:** Echezona E. Ezeanolue, Theddeus Iheanacho, Dina V. Patel, Shatabdi Patel, Nadia Sam-Agudu, Michael Obiefune, Patrick Dakum, Prosper Okonkwo, Ayodotun Olutola, Hadiza Khamofu, Bolanle Oyeledun, Sani Aliyu, Muyiwa Aina, Andy Eyo, John Oko, Timothy Akinmurele, Obinna Oleribe, Usman Gebi, Muktar H. Aliyu, Rachel Sturke, George Siberry

**Affiliations:** 1 Department of Pediatrics and Child Health, University of Nigeria, Enugu-Campus, NG; 2 Healthy Sunrise Foundation, Las Vegas, NV, US; 3 Yale University, New Haven Connecticut, US; 4 Institute of Human Virology, Abuja, NG; 5 Institute of Human Virology, University of Maryland, Baltimore, MD, US; 6 AIDS Prevention Initiative, Abuja, NG; 7 Centre for Clinical Care and Clinical Research, Abuja, NG; 8 Family Health International, Abuja, NG; 9 Center for Integrated Health Programs, Abuja, NG; 10 National Agency for the Control of AIDS, Abuja, NG; 11 Solina Health, Abuja, NG; 12 Excellence Community Education Welfare Scheme, Abuja, NG; 13 Catholic Caritas Foundation Nigeria, Abuja, NG; 14 Enhanced Health Access Initiatives, Abuja, NG; 15 Excellence and Friends Management Consult, Abuja, NG; 16 Friends for Global Health Initiative, NG; 17 Equitable Health Access Initiative, NG; 18 Vanderbilt Institute for Global Health, Vanderbilt University Medical Center, Nashville, TN, US; 19 Fogarty International Center, National Institutes of Health, Bethesda, MD, US; 20 United States Agency for International Development, Washington DC, US

## Abstract

**Background:**

Contextual research evidence is needed to reduce morbidity and mortality due to chronic but preventable diseases in low- and middle-income countries. Nigeria, Africa’s most populous country, is particularly burdened by these diseases despite its academic and research infrastructure. A major impediment to developing robust evidence on sustainable disease prevention and treatment strategies is the lack of skilled research personnel.

**Objective:**

This study aimed to identify (1) training barriers for research assistants and coordinators and (2) potential strategies to counter these barriers using a Nominal Group Technique (NGT) exercise conducted at the 2017 conference of the Nigeria Implementation Science Alliance (NISA).

**Method:**

A one-hour NGT exercise was conducted with 26 groups of 2–9 persons each (N = 134) drawn from conference attendees. Group members were presented with questions related to the two objectives. Each member was asked to generate, list, discuss and vote on ideas that were eventually ranked by the group. Qualitative Thematic Analysis (QTA) was conducted for the collated responses.

**Findings:**

The QTA identified 166 training gaps and 147 potential solutions, out of which 104 were ranked. Themes that emerged for gaps included: 1) inadequate mentorship; 2) inadequate training/lack of organized curriculum; 3) limited access to opportunities for training and employment; 4) lack of government funding; 5) lack of interest, motivation; and 6) lack of research culture. Themes for potential strategies to address the gaps were: 1) trainings/curriculum development; 2) research modules implemented in secondary and tertiary institutions; 3) creating a sustainable forum for research-related questions and answers; and 4) advocating for and accessing more government funding for research training.

**Conclusion:**

This study identified actionable strategies that reflect practical realities in implementation research in Nigeria, which can guide government agencies, policy makers, research organizations, and local foundations as they work together to increase research capacity in Nigeria.

## Introduction

Biomedical research emanating from low- and middle-income countries (LMICs), especially in sub-Saharan Africa, remains disproportionately low despite recent growth in global health research [1]. Published estimates suggest that only about 1% of biomedical publications originate from Africa [2,3]. Given that sub-Saharan African countries and other LMICs still carry the highest burden of morbidity and mortality due to preventable and treatable diseases [4], there is a critical need to enhance local and sustainable research expertise geared towards addressing the unique challenges to disease prevention and treatment in these countries. Nigeria, Africa’s most populous country, has a relatively more robust academic infrastructure than many other sub-Saharan countries [5,6]. Additionally, Nigeria has many donor-sponsored clinical and implementation programs targeting infectious diseases such as HIV, tuberculosis, and malaria [7]. Yet, despite this, Nigeria’s national health indices show persistently poor outcomes. Specifically, there remains a high level of maternal and child mortality, rising incidence of non-communicable diseases, limited access to mental health care, and little or no public or private health insurance coverage for most of the population [8,9,10]. Until recently, little effort had been expended to leverage existing clinical and implementation programs to increase locally impactful research capacity, collaboration between programs and academia, and linkage of research evidence to policy [11,12]. A deliberate, robust, and sustained approach to building local scientific research infrastructure is needed to support evidence-based policy making and program implementation using locally relevant data [13]. Indeed, Nigeria’s National Strategic Health Development Plan identified research capacity strengthening as one of the eight priority areas for utilizing research to inform policy and programs, improve health, and contribute to the global knowledge platform [14,15,16].

Key challenges limiting research capacity-building in Nigeria and other LMICs are: a) few qualified researchers and trained support staff, including research assistants and research coordinators; b) poor research infrastructure, such as low access to computers, laboratories, and broadband internet access; c) lack of expertise in grant writing, manuscript preparation, and access to international published works; and d) disproportionately low funding priority for research by governments and institutions [17,18,19,20].

The Nigeria Implementation Science Alliance (NISA) was established in 2015 as a partnership of 20 organizations that included academic and non-governmental organizations, clinical service providers, and policy makers. NISA is focused on facilitating collaboration among partners, enhancing implementation research in Nigeria and the sub-Saharan region, and identifying feasible, culturally appropriate strategies to improve public health through research [11,21].

In September 2017, the third NISA scientific conference was held in Abuja, Nigeria. The conference focused on identifying ways to promote implementation research by building research capacity through training. During the conference, researchers, policy makers, and public health program implementers from Nigeria examined key challenges facing the country in the area of locally available, competent research personnel and possible strategies to bridge existing gaps. This paper documents findings from this group session and provides guidance towards creating a sustainable pool of mid-level research personnel, namely research assistants and coordinators to support a thriving research community.

## Methods

### Process and participants

A one-hour structured modified Nominal Group Technique (NGT) exercise [22] was conducted to identify and prioritize (1) training barriers for research assistants and coordinators, and (2) potential strategies to address the barriers identified. One hundred thirty-four individuals participated in this NGT in 26 groups of 2 to 9 individuals each (average group size = 6 individuals). Group members included representatives from HIV/AIDS program implementing partners funded by the U.S. President’s Emergency Plan for AIDS Relief (PEPFAR), academia, researchers, clinicians, and policy makers. Among the 134 participants in this NGT exercise, 65 (48%) identified as program implementing partners, 16 (12%) as policy makers, and 18 (13%) as researchers. Some participants identified as belonging to multiple categories, either as implementing partner and researcher, 25 (19%), implementing partner, researcher, and policy maker, 5 (4%), researcher and policy maker, 2 (1.5%), or implementing partner and policy maker, 1 (0.8%) (Table 1).

**Table 1:** Nominal Group Technique Participants’ Self-identified roles.

Group (N = 134)	Number (%)	

Implementing partners (IP)	65	(%)
Research institutions	18	
Policy-makers	16	
IP and researchers	25	
IP and policy-makers	1	
Researchers and policy-makers	2	
IP, researchers and policy-makers	5	
Other	2	

The Nominal Group Technique (NGT) was conducted in two 30-minute phases, with three distinct components designed to maximize participant focus and engagement. For the first 30 minutes, each group member identified challenges and barriers in training research assistants and coordinators in Nigeria, followed by a group discussion that elaborated on the identified barriers. The second 30-minute phase focused on identifying strategies to address the challenges and barriers identified in the previous session. The second phase also involved each group member identifying potential solutions. Each of these 30-minute sessions included three distinct activities, namely (1) generating ideas (i.e. challenges or barriers), (2) listing these ideas, and (3) ranking the perceived barriers and potential solutions identified in the first two steps. Utilizing “brainstorming”, voting and ranking of items as part of this NGT is an effective, efficient and low-cost method of identifying multiple barriers and potential remedies from persons actively involved in implementation projects in Nigeria [11,21,23]. After generating a list of items, groups were asked to rank the top four items by order of perceived importance in each category for both perceived barriers and potential solutions. At the end of the NGT exercise, each group selected a representative to share their group’s top-ranked responses in each category with the rest of the participant groups. After the exercise, all the items ranked by the 26 groups were collected, collated, and later transcribed for data analysis and interpretation.

## Analysis

First, all responses were transcribed and entered into an Excel® datasheet and sorted to identify potential duplicate or invalid entries. Statements were reviewed and eliminated if confirmed as duplicate or invalid. All valid responses were collated and sorted and then combined based on recurring themes [24]. For each barrier and solution that could be explained by more than two themes, the two themes that most strongly captured the idea were included. Next, themes of identified barriers to training research assistants and coordinators and potential solutions were ranked in order of importance by the participants. All data collection documents were de-identified prior to analysis. This study was approved by the Nigerian National Health Research Ethics Committee.

## Results

Each of the 26 NGT groups independently identified between 4 and 9 barriers and between 3 and 8 potential solutions. Ultimately, the exercise yielded a total of 166 barriers with 104 ranked items and 147 solutions also with 104 ranked items. There were six coalescing themes from the list of barriers and six themes from the list of potential solutions (Table 2). Themes under barriers reflected inadequate mentorship, training, research opportunities, funding, motivation, and research culture. The specific points made by each category of stakeholders reflected their unique circumstances as it related to the identified barriers. Some of the identified barriers limiting the availability of trained research assistants and coordinators included “principal investigators and seniors (more experienced members of research teams) do not invest time and other resources in training research assistants and coordinators”, “weak capacity and lack of enabling environment for strong mentorship”, “inadequate manpower to train research assistants and coordinators”, and “not a recognized role in health service system”.

**Table 2:** Coalesced themes of training barriers and suggested solutions.

Barriers	Solutions

1. Poor mentorship	Increase opportunity to identify mentors (support conference attendance, like NISA)
2. Inadequate training/lack of organized curriculum	Research module associated with institutions; create a forum for research related questions
3. Inaccessible opportunities	Training/curriculum led by NISA, including appropriate research curriculum in educational settings
4. Lack of government funding	Increase government funding; advocate for higher appropriations for research, dedicate specific funds in grants for training research assistants
5. Lack of interest and motivation	Increase pay for research assistants and coordinator positions to attract interest, create awareness of a potential career path for RA and RC positions, make training opportunities more flexible example: use online webinars
6. Lack of research culture	Use of social media to highlight research findings in Nigeria, more collaboration between research/academic institutions and non-academic/research healthcare organizations

The six themes that captured potential ways of overcoming the identified barriers were mentor-mentee networking, research curriculum, training opportunities, dedicated and protected funding for research training, incentives for research assistant and coordinator positions, and the use of social media to disseminate research findings in Nigeria (Table 2). Specific examples under these themes were “training and mentorship program for trainees” “make research more attractive”, “advocacy to have research assistants and coordinators recognized as a career pathway, job designation”, “develop a standard training curriculum in universities”, and “appropriate resource mobilization and funding”. These identified barriers and suggested solutions represent a variety of different perspectives of the participants – implementers, researchers, or policy makers – and thus spanned various structural frameworks including government policy, educational system, training, and research programs.

Table 3 displays results of participant ranking of barriers and potential solutions in order of importance. The top-ranked barrier by importance was the perception by program staff that “research was not their primary job”. The top ranked solution was “inclusion of appropriate research curriculum at all levels of education”.

**Table 3:** Top-ranked barriers and solutions by order of importance.

Top Barriers	Top Solutions

1. Research Assistants and Coordinators feeling overwhelmed because research is not their primary job	Include appropriate research curriculum at all levels of education
2. Research seems inaccessible especially to young people and outside academic settings	Increase funding for research assistant positions
3. Inadequate funding	Increase pay for Research Assistants and Coordinators positions
4. Defining qualification and selection process for research assistants/coordinators	Create awareness and advocacy for need for research assistants and coordinators

## Discussion

This Nominal Group Technique exercise and the subsequent analysis elucidated 12 key themes regarding barriers to and strategies for increased training for research assistants and coordinators in Nigeria. The six themes that emerged relating to barriers (inadequate mentorship, limited training and research opportunities, poor funding, low motivation, and absence of a research culture in most academic institutions) are consistent with findings from other LMICs [25]. Six themes also emerged regarding strategies for enhanced training, namely: mentorship, a formal research curriculum in schools, training opportunities for staff, protected funding for research training, incentivizing research assistant and coordinator positions, and adequately disseminating research findings in Nigeria. These barriers and strategies can be better understood in the context of overall healthcare investment in Nigeria, the nature of public-private partnerships that promote health research, sustainability of donor-driven program implementation, and the structure of research frameworks established by existing legislation [26].

A key strength of this study is that participants include multi-disciplinary frontline staff with an average of 10 years of local field experience in implementing clinical and research projects in Nigeria, unlike previously published data which elicited responses only from academic center-based researchers [27]. Although we did not ask specifically for the nationality of attendees, we collected information on primary place of work which indicate that majority of respondents work primarily in Nigeria. In addition, instead of focusing on principal investigators, this study focuses on study implementation personnel (research assistants and research coordinators) who conduct fieldwork and research administration. Therefore, most participants had first-hand knowledge and experience about the context of program implementation, the role of government and funding agencies, the policy framework for the programs, and the funding mechanisms available in Nigeria. In addition, utilizing the group process in this study allowed for brainstorming that enabled interaction of multiple perspectives and the generation of tangible ideas. Further, in coming together under NISA’s umbrella organizations and partner government departments (the Federal Ministry of Health, the National Primary Healthcare Development Agency, and the National Agency for the Control of AIDS), we have taken advantage of the strong working relationships that we have helped establish among key stakeholders in academia, government, donor/funding agencies, and program implementers [11]. This is a significant first step towards implementing some of the capacity-building strategies identified in this NGT. NISA and similar alliances can leverage available research, education, and policy opportunities to provide a platform for connecting research mentors and mentees, training opportunities, and programs with collaborating universities and research institutions, as well as policy frameworks that are favorable for successful research career pathways. By collecting implementation data, reviewing outcomes, and performing cost-effectiveness analysis, this community of researchers and implementation programs within NISA can articulate the economic benefits and cost-saving opportunities of health programs, and make the case for more downstream investment in research infrastructures in schools, colleges, and universities.

Recently, following activities with NISA, including conferences and deliberations, various organizations and government agencies have made definite commitments toward more investment in implementation research in Nigeria. For example, the National Agency for the Control of AIDS (NACA) in collaboration with the United Nations Children’s Fund (UNICEF) and the Population Council this year launched a pilot project called “Adolescent and Young People Challenge” that seeks to fund innovative ideas led by youth to provide comprehensive HIV education to at least 200,000 Nigerians. This type of initiative can accomplish the broader goal of HIV/AIDS awareness but also increase interest in health care and implementation research among young Nigerian trainees and professionals while matching them with appropriate research mentors. Another example is the Healthy Sunrise Foundation (HSF), a non-profit, non-government organization that has held grant-writing workshops in Nigeria for research assistants and coordinators working with implementation programs [28]. HSF also supports scale up of evidence-based interventions like the Baby Shower Initiative [29]. These types of initiatives can accomplish the broader goal of HIV/AIDS awareness and expansion of access to care while also increasing interest in implementation research and matching participants with appropriate research mentors.

As the NISA platform expands and strengthens, it is important to have greater involvement of in-country academic institutions in understanding the challenges and opportunities for training and employing research assistants and coordinators. This is important because academia has a critical role to play in capacity building for research support staff as well as employing them after their formal training. The future for this looks promising as key universities in Nigeria like University of Nigeria, University of Ibadan, Ahmadu Bello University, and University of Jos have been represented in recent NISA conferences.

This study has some limitations. First, the NGT, unlike a free-flowing focus group, has a rigid format and is time limited. To minimize the impact of this limitation, many participants with varying experiences in research were included, thereby ensuring that the perspectives, ideas, and emergent consensus were as representative as possible. Second, as in other group-based research, participants’ expressed opinions and suggestions may have been influenced by others in the group. To reduce the impact of this group factor, we created many small groups instead of a few larger groups. Finally, the authors conducted the analyses and thus may have inadvertently infused their own perspectives in interpreting the data. To minimize this, we have included authors from a wide variety of backgrounds and experiences to ensure balanced perspectives in the analysis and interpretation of the results.

## Conclusion

Skilled research coordinators and research assistants are the center of the research enterprise and directly responsible for ensuring that research is performed ethically and in accordance with study requirements. In short, they are the lifeline of high-quality, impactful research work [30,31]. The barriers and potential solutions that emerged from this study reflect the lived experiences, practical realities, and identifiable potential of mid-level research personnel involved in implementation research in Nigeria. Our findings serve as a useful guide for government agencies, policy makers, research organizations, and local foundations as they work together to increase research capacity in Nigeria.
